# Crystal structure of 2-[2-(hy­droxy­imino)-1-phenyl­propyl­idene]-*N*-phen­ylhydrazinecarbo­thio­amide

**DOI:** 10.1107/S2056989015017739

**Published:** 2015-09-26

**Authors:** Brian J. Anderson, Michael B. Freedman, Sean P. Millikan, Victoria A. Smolenski, Jerry P. Jasinski

**Affiliations:** a Department of Chemistry, Keene State College, 229 Main Street, Keene, NH 03435-2001, USA

**Keywords:** crystal structure, thio­semicarbazone, weak inter­molecular inter­actions, O—H⋯π inter­actions

## Abstract

In the title compound, C_16_H_16_N_4_OS, an intra­molecular C—H⋯S hydrogen bond is observed. With the exception of the phenyl ring of the phenyl­propyl­idene unit, the remainder of the mol­ecule has an almost planar skeleton with an r.m.s. deviation of 0.121 (5) Å from the plane through the remaining 16 atoms. In the crystal O—H⋯N hydrogen bonds are observed between the terminal hy­droxy­imino groups, forming inverson dimers with *R*_2_^2^(6) graph-set motifs. Additional C—H⋯N contacts stack the dimers along [100]. While no π—π inter­actions are present, weak C—H⋯O and O—H⋯*Cg* inter­actions are also observed and help stabilize the crystal packing.

## Related literature   

For thio­semicarbazone ligands and their metal complexes, see: Lobana *et al.* (2009 ▸, 2012 ▸). For the biological, anti-tumor and anti-fungal activity of palladium complexes with thio­semicarbazone ligands, see: Chellan *et al.* (2010 ▸). For the biological activity of a thio­semicarbazone ligand with terminal dimethyl substitution, see: Kowol *et al.* (2009 ▸). For related structures, see Anderson *et al.* (2012 ▸, 2013 ▸).
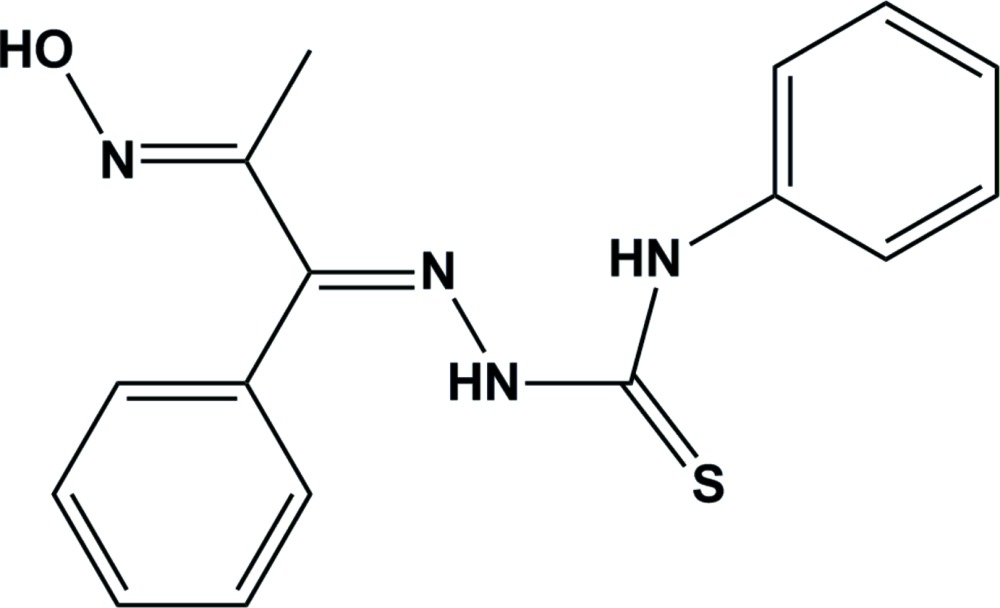


## Experimental   

### Crystal data  

C_16_H_16_N_4_OS
*M* *_r_* = 312.39
Monoclinic, 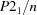

*a* = 5.4955 (6) Å
*b* = 27.973 (2) Å
*c* = 10.4175 (9) Å
β = 92.444 (9)°
*V* = 1600.0 (3) Å^3^
*Z* = 4
Mo *K*α radiation
μ = 0.21 mm^−1^
*T* = 173 K
0.42 × 0.14 × 0.08 mm

### Data collection  

Agilent Eos, Gemini diffractometer
Absorption correction: multi-scan (*CrysAlis PRO*; Agilent, 2014 ▸) *T*_min_ = 0.747, *T*_max_ = 1.000
17587 measured reflections
5402 independent reflections
4006 reflections with *I* > 2σ(*I*)
*R*_int_ = 0.044

### Refinement  

*R*[*F* ^2^ > 2σ(*F* ^2^)] = 0.065
*wR*(*F* ^2^) = 0.182
*S* = 1.04
5402 reflections
201 parameters
H-atom parameters constrained
Δρ_max_ = 0.56 e Å^−3^
Δρ_min_ = −0.37 e Å^−3^

### 

Data collection: *CrysAlis PRO* (Agilent, 2014 ▸); cell refinement: *CrysAlis PRO*; data reduction: *CrysAlis RED* (Agilent, 2014 ▸); program(s) used to solve structure: *SHELXT* (Sheldrick, 2015*a* ▸); program(s) used to refine structure: *SHELXL* (Sheldrick, 2015*b* ▸); molecular graphics: *OLEX2* (Dolomanov *et al.*, 2009 ▸); software used to prepare material for publication: *OLEX2*.

## Supplementary Material

Crystal structure: contains datablock(s) global, I. DOI: 10.1107/S2056989015017739/sj5476sup1.cif

Structure factors: contains datablock(s) I. DOI: 10.1107/S2056989015017739/sj5476Isup2.hkl

Supporting information file. DOI: 10.1107/S2056989015017739/sj5476Isup3.cml

**Fig. 1.**
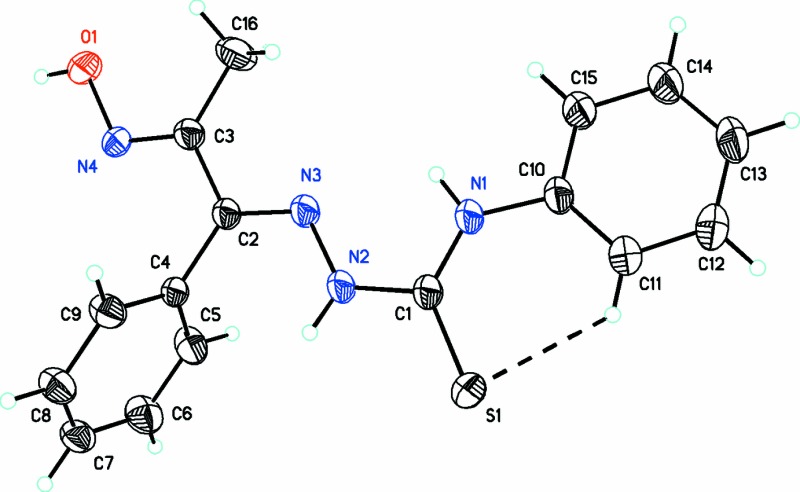
The molecular structure of C16H16N4OS, (I), showing the atom-labelling scheme. Displacement ellipsoids are drawn at the 30% probability level. The dashed line indicates a weak C11—H11···S1 intramolecular contact.

16 16 4 . DOI: 10.1107/S2056989015017739/sj5476fig1.tif
The mol­ecular structure of C_16_H_16_N_4_OS, (I), showing the atom-labelling scheme. Displacement ellipsoids are drawn at the 30% probability level. The dashed line indicates a weak C11—H11⋯S1 intra­molecular contact.

**Fig. 2.**
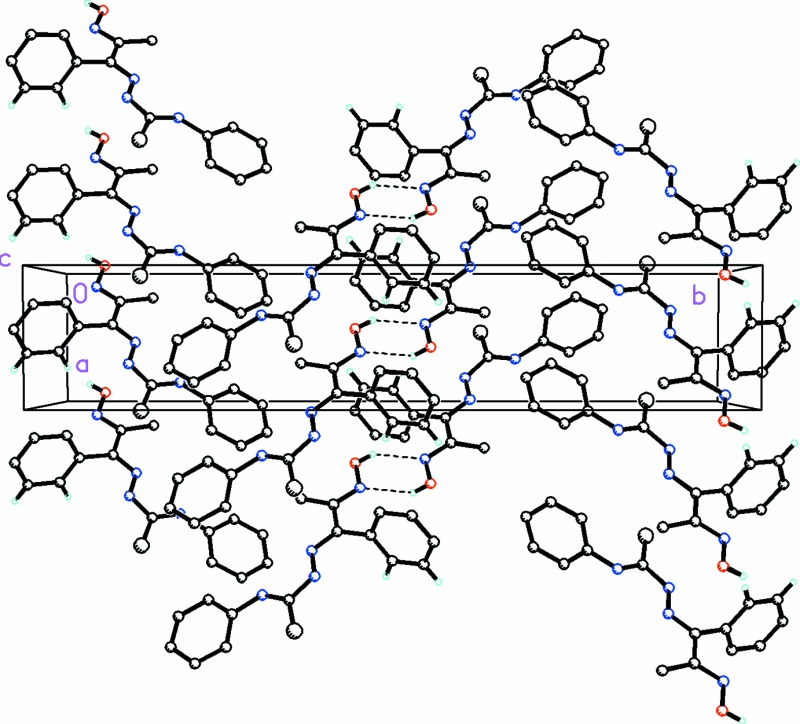
Packing diagram of (I) viewed along the c axis. Dashed lines indicate O—H—N hydrogen bonds between the terminal hydroxy amino groups forming R22(6) inverson dimers stacked along [1 0 0]. The H atoms not involved in these interactions have been omitted for clarity.

Click here for additional data file.c . DOI: 10.1107/S2056989015017739/sj5476fig2.tif
Packing diagram of (I) viewed along the *c* axis. Dashed lines indicate O—H—N hydrogen bonds between the terminal hy­droxy amino groups forming 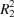
(6) inverson dimers stacked along [1 0 0]. The H atoms not involved in these inter­actions have been omitted for clarity.

CCDC reference: 1426205

Additional supporting information:  crystallographic information; 3D view; checkCIF report

## Figures and Tables

**Table 1:** Hydrogen-bond geometry (, ) *Cg*1 is the centroid of the C4C9 phenyl ring.

*D*H*A*	*D*H	H*A*	*D* *A*	*D*H*A*
O1H1N4^i^	0.82	2.20	2.867(2)	139
C5H5O1^ii^	0.93	2.84	3.451(3)	124
C5H5N4^ii^	0.93	2.85	3.472(2)	125
C6H6O1^ii^	0.93	2.82	3.442(3)	125
C11H11S1	0.93	2.54	3.193(2)	127
O1H1*Cg*1^i^		2.78	3.3309(17)	126

Symmetry codes: (i) 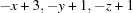; (ii) 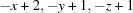.

## supplementary crystallographic information

### S1. Comment

In the title compound, C_16_H_16_N_4_OS, (I), one independent molecule crystallizes in the asymmetric unit and forms an intramolecular C11—H11···S1 hydrogen bond, (Fig. 1). With the exception of the C4···.C9 phenyl ring of the phenylpropylidene unit, the remainder of the molecule has an almost planar skeleton with an *rms* deviation of 0.121 (5) Å from the plane through the remaining 16 atoms. In the crystal O1—H1···N4 hydrogen bonds are observed between the terminal hydroxyimino groups forming inverson dimers with *R*_2_^2^(6) graph-set motifs (Table 1) Additional C5—H5···N4 contacts stack the dimers along [1 0 0]. While no π–π interactions are present, weak C5—H5···O1, C6—H6···O1 and O1—H1···π interactions are also observed, and help stablize the crystal packing.

### S2. Experimental

In a 25 ml round bottom flask 0.205 g (1.26 mmol) of 1-phenyl-1, 2-propanedione 2-oxine and 0.211 g (1.26 mmol) of 4-phenylthiosemicarbazide were dissolved in 20 ml of methanol. One drop of sulfuric acid was added to catalyze the reaction. The resulting clear solution was refluxed for 12 h and there was a noticeable yellow color change. The reaction was removed from the heat and cooled to room temperature. The resulting yellow solution was transferred to a 125 ml separatory funnel. Dichloromethane (10 ml) and water (10 ml) were added, and the organic layer was separated. The aqueous layer was extracted with an additional 10 ml of dichloromethane, and then the organic layers were combined, washed with brine (2 *x* 10 ml), dried with magnesium sulfate, and the solvent was removed *in vacuo* to give a yellow solid. The product was recrystallized from hot acetonitrile. m.p. 463–464 K.

### S3. Refinement

Crystal data, data collection and structure refinement details are summarizedin Table 1. A l l H atoms were located in difference maps. The C–H and N–H atoms were treated as riding atoms in geometrically idealized positions with C—H, N—H distances of 0.93 Å, 0.86 Å and refined with *U*_iso_(H) = 1.2*U*_eq_(C, N). The CH_3_ and O–H atoms were also treated as riding atoms in geometrically idealized positions with the CH_3_, O—H distances of 0.96 Å, 0.84 Å and refined with *U*_iso_(H) = 1.5*U*_eq_(C, O).

### Crystal data

**Table:**

C_16_H_16_N_4_OS	*F*(000) = 656
*M**_r_* = 312.39	*D*_x_ = 1.297 Mg m^−^^3^
Monoclinic, *P*2_1_/*n*	Mo *K*α radiation, λ = 0.71073 Å
*a* = 5.4955 (6) Å	Cell parameters from 4571 reflections
*b* = 27.973 (2) Å	θ = 3.5–32.9°
*c* = 10.4175 (9) Å	µ = 0.21 mm^−^^1^
β = 92.444 (9)°	*T* = 173 K
*V* = 1600.0 (3) Å^3^	Needle, colourless
*Z* = 4	0.42 × 0.14 × 0.08 mm

### Data collection

**Table:**

Agilent Eos, Gemini diffractometer	5402 independent reflections
Radiation source: Enhance (Mo) X-ray Source	4006 reflections with *I* > 2σ(*I*)
Graphite monochromator	*R*_int_ = 0.044
Detector resolution: 16.0416 pixels mm^-1^	θ_max_ = 33.0°, θ_min_ = 3.5°
ω scans	*h* = −7→6
Absorption correction: multi-scan (*CrysAlis PRO*; Agilent, 2014)	*k* = −39→42
*T*_min_ = 0.747, *T*_max_ = 1.000	*l* = −14→15
17587 measured reflections	

### Refinement

**Table:**

Refinement on *F*^2^	Primary atom site location: structure-invariant direct methods
Least-squares matrix: full	Hydrogen site location: inferred from neighbouring sites
*R*[*F*^2^ > 2σ(*F*^2^)] = 0.065	H-atom parameters constrained
*wR*(*F*^2^) = 0.182	*w* = 1/[σ^2^(*F*_o_^2^) + (0.0835*P*)^2^ + 0.6623*P*] where *P* = (*F*_o_^2^ + 2*F*_c_^2^)/3
*S* = 1.04	(Δ/σ)_max_ = 0.001
5402 reflections	Δρ_max_ = 0.56 e Å^−^^3^
201 parameters	Δρ_min_ = −0.37 e Å^−^^3^
0 restraints	

### Special details

**Table:**

Geometry. All e.s.d.'s (except the e.s.d. in the dihedral angle between two l.s. planes) are estimated using the full covariance matrix. The cell e.s.d.'s are taken into account individually in the estimation of e.s.d.'s in distances, angles and torsion angles; correlations between e.s.d.'s in cell parameters are only used when they are defined by crystal symmetry. An approximate (isotropic) treatment of cell e.s.d.'s is used for estimating e.s.d.'s involving l.s. planes.

### Fractional atomic coordinates and isotropic or equivalent isotropic displacement parameters (Å^2^)

**Table:**

	*x*	*y*	*z*	*U*_iso_*/*U*_eq_	
S1	0.45822 (11)	0.63428 (2)	0.03118 (5)	0.04249 (17)	
O1	1.5672 (3)	0.55466 (5)	0.56408 (13)	0.0352 (3)	
H1	1.6317	0.5292	0.5847	0.053*	
N1	0.6605 (3)	0.68290 (6)	0.23282 (16)	0.0396 (4)	
H1A	0.7732	0.6814	0.2925	0.047*	
N2	0.8045 (3)	0.60886 (5)	0.19247 (14)	0.0317 (3)	
H2	0.8036	0.5826	0.1497	0.038*	
N3	0.9654 (3)	0.61454 (5)	0.29421 (14)	0.0292 (3)	
N4	1.3985 (3)	0.54711 (5)	0.46248 (13)	0.0261 (3)	
C1	0.6445 (3)	0.64429 (6)	0.15726 (16)	0.0283 (3)	
C2	1.1020 (3)	0.57835 (5)	0.32323 (15)	0.0248 (3)	
C3	1.2802 (3)	0.58508 (6)	0.43056 (16)	0.0275 (3)	
C4	1.0875 (3)	0.53131 (5)	0.25667 (14)	0.0234 (3)	
C5	0.8957 (4)	0.50060 (6)	0.27837 (19)	0.0351 (4)	
H5	0.7749	0.5097	0.3333	0.042*	
C6	0.8837 (4)	0.45653 (7)	0.2185 (2)	0.0398 (4)	
H6	0.7553	0.4359	0.2335	0.048*	
C7	1.0618 (4)	0.44308 (6)	0.13634 (18)	0.0327 (4)	
H7	1.0540	0.4133	0.0968	0.039*	
C8	1.2505 (4)	0.47357 (7)	0.11285 (18)	0.0344 (4)	
H8	1.3697	0.4645	0.0570	0.041*	
C9	1.2637 (4)	0.51787 (6)	0.17228 (17)	0.0311 (4)	
H9	1.3908	0.5386	0.1556	0.037*	
C10	0.5243 (4)	0.72573 (6)	0.23153 (18)	0.0365 (4)	
C11	0.3061 (6)	0.73214 (9)	0.1637 (3)	0.0676 (9)	
H11	0.2395	0.7075	0.1136	0.081*	
C12	0.1862 (6)	0.77580 (10)	0.1708 (3)	0.0749 (10)	
H12	0.0384	0.7801	0.1256	0.090*	
C13	0.2824 (6)	0.81243 (9)	0.2433 (3)	0.0648 (8)	
H13	0.2094	0.8424	0.2422	0.078*	
C14	0.4845 (7)	0.80392 (10)	0.3160 (4)	0.0897 (14)	
H14	0.5435	0.8275	0.3721	0.108*	
C15	0.6072 (6)	0.76113 (9)	0.3097 (3)	0.0695 (10)	
H15	0.7492	0.7566	0.3601	0.083*	
C16	1.3123 (5)	0.63254 (7)	0.4947 (2)	0.0491 (6)	
H16A	1.1963	0.6358	0.5605	0.074*	
H16B	1.2872	0.6575	0.4323	0.074*	
H16C	1.4743	0.6348	0.5325	0.074*	

### Atomic displacement parameters (Å^2^)

**Table:**

	*U*^11^	*U*^22^	*U*^33^	*U*^12^	*U*^13^	*U*^23^
S1	0.0480 (3)	0.0359 (3)	0.0417 (3)	0.0114 (2)	−0.0199 (2)	−0.00529 (18)
O1	0.0361 (7)	0.0355 (7)	0.0326 (6)	0.0052 (5)	−0.0142 (5)	−0.0002 (5)
N1	0.0474 (10)	0.0274 (8)	0.0420 (9)	0.0158 (7)	−0.0197 (7)	−0.0071 (6)
N2	0.0390 (9)	0.0235 (7)	0.0316 (7)	0.0117 (6)	−0.0107 (6)	−0.0037 (5)
N3	0.0349 (8)	0.0248 (7)	0.0272 (6)	0.0086 (6)	−0.0069 (5)	−0.0020 (5)
N4	0.0256 (7)	0.0276 (7)	0.0244 (6)	0.0044 (5)	−0.0055 (5)	−0.0006 (5)
C1	0.0322 (9)	0.0226 (7)	0.0297 (8)	0.0055 (6)	−0.0040 (6)	0.0024 (6)
C2	0.0273 (8)	0.0221 (7)	0.0248 (7)	0.0046 (6)	−0.0024 (6)	0.0000 (5)
C3	0.0311 (9)	0.0240 (7)	0.0271 (7)	0.0032 (6)	−0.0035 (6)	−0.0012 (5)
C4	0.0256 (8)	0.0206 (7)	0.0236 (7)	0.0045 (5)	−0.0037 (5)	0.0002 (5)
C5	0.0335 (10)	0.0273 (8)	0.0453 (10)	0.0007 (7)	0.0102 (7)	−0.0021 (7)
C6	0.0398 (11)	0.0264 (9)	0.0535 (11)	−0.0069 (7)	0.0074 (9)	−0.0015 (8)
C7	0.0405 (10)	0.0221 (7)	0.0349 (8)	0.0011 (6)	−0.0036 (7)	−0.0030 (6)
C8	0.0383 (10)	0.0321 (9)	0.0332 (8)	0.0006 (7)	0.0059 (7)	−0.0070 (6)
C9	0.0320 (9)	0.0286 (8)	0.0332 (8)	−0.0041 (6)	0.0059 (7)	−0.0051 (6)
C10	0.0435 (11)	0.0274 (8)	0.0375 (9)	0.0130 (7)	−0.0100 (8)	−0.0034 (6)
C11	0.0659 (18)	0.0420 (12)	0.091 (2)	0.0249 (12)	−0.0401 (15)	−0.0243 (12)
C12	0.074 (2)	0.0538 (15)	0.093 (2)	0.0350 (14)	−0.0419 (17)	−0.0214 (14)
C13	0.078 (2)	0.0420 (12)	0.0712 (16)	0.0336 (13)	−0.0279 (14)	−0.0210 (11)
C14	0.103 (3)	0.0505 (15)	0.109 (3)	0.0422 (16)	−0.066 (2)	−0.0462 (16)
C15	0.078 (2)	0.0453 (13)	0.0806 (18)	0.0310 (13)	−0.0487 (15)	−0.0319 (12)
C16	0.0690 (16)	0.0278 (9)	0.0481 (12)	0.0066 (9)	−0.0249 (11)	−0.0100 (8)

### Geometric parameters (Å, º)

**Table:**

S1—C1	1.6543 (18)	C7—H7	0.9300
O1—H1	0.8200	C7—C8	1.373 (3)
O1—N4	1.3930 (17)	C8—H8	0.9300
N1—H1A	0.8600	C8—C9	1.386 (2)
N1—C1	1.337 (2)	C9—H9	0.9300
N1—C10	1.412 (2)	C10—C11	1.377 (3)
N2—H2	0.8600	C10—C15	1.349 (3)
N2—N3	1.3604 (19)	C11—H11	0.9300
N2—C1	1.365 (2)	C11—C12	1.391 (3)
N3—C2	1.288 (2)	C12—H12	0.9300
N4—C3	1.282 (2)	C12—C13	1.366 (4)
C2—C3	1.467 (2)	C13—H13	0.9300
C2—C4	1.488 (2)	C13—C14	1.339 (4)
C3—C16	1.493 (2)	C14—H14	0.9300
C4—C5	1.386 (2)	C14—C15	1.377 (3)
C4—C9	1.387 (2)	C15—H15	0.9300
C5—H5	0.9300	C16—H16A	0.9600
C5—C6	1.382 (3)	C16—H16B	0.9600
C6—H6	0.9300	C16—H16C	0.9600
C6—C7	1.379 (3)		
			
N4—O1—H1	109.5	C7—C8—H8	120.0
C1—N1—H1A	114.4	C7—C8—C9	120.09 (18)
C1—N1—C10	131.15 (15)	C9—C8—H8	120.0
C10—N1—H1A	114.4	C4—C9—H9	120.0
N3—N2—H2	119.5	C8—C9—C4	120.06 (17)
N3—N2—C1	120.97 (14)	C8—C9—H9	120.0
C1—N2—H2	119.5	C11—C10—N1	124.38 (18)
C2—N3—N2	116.34 (14)	C15—C10—N1	116.84 (18)
C3—N4—O1	112.67 (13)	C15—C10—C11	118.60 (19)
N1—C1—S1	128.83 (13)	C10—C11—H11	120.3
N1—C1—N2	113.76 (15)	C10—C11—C12	119.4 (2)
N2—C1—S1	117.40 (13)	C12—C11—H11	120.3
N3—C2—C3	116.19 (14)	C11—C12—H12	119.5
N3—C2—C4	124.51 (14)	C13—C12—C11	121.0 (2)
C3—C2—C4	119.30 (13)	C13—C12—H12	119.5
N4—C3—C2	113.98 (14)	C12—C13—H13	120.9
N4—C3—C16	124.84 (16)	C14—C13—C12	118.1 (2)
C2—C3—C16	121.17 (15)	C14—C13—H13	120.9
C5—C4—C2	119.88 (15)	C13—C14—H14	119.2
C5—C4—C9	119.45 (15)	C13—C14—C15	121.6 (2)
C9—C4—C2	120.67 (15)	C15—C14—H14	119.2
C4—C5—H5	120.0	C10—C15—C14	120.9 (2)
C6—C5—C4	120.09 (17)	C10—C15—H15	119.6
C6—C5—H5	120.0	C14—C15—H15	119.6
C5—C6—H6	119.9	C3—C16—H16A	109.5
C7—C6—C5	120.12 (18)	C3—C16—H16B	109.5
C7—C6—H6	119.9	C3—C16—H16C	109.5
C6—C7—H7	119.9	H16A—C16—H16B	109.5
C8—C7—C6	120.17 (16)	H16A—C16—H16C	109.5
C8—C7—H7	119.9	H16B—C16—H16C	109.5
			
O1—N4—C3—C2	179.66 (14)	C3—C2—C4—C9	−75.1 (2)
O1—N4—C3—C16	−0.9 (3)	C4—C2—C3—N4	−4.6 (2)
N1—C10—C11—C12	179.2 (3)	C4—C2—C3—C16	176.00 (19)
N1—C10—C15—C14	−179.2 (3)	C4—C5—C6—C7	−0.4 (3)
N2—N3—C2—C3	178.26 (15)	C5—C4—C9—C8	−1.6 (3)
N2—N3—C2—C4	−2.4 (3)	C5—C6—C7—C8	−0.6 (3)
N3—N2—C1—S1	179.26 (14)	C6—C7—C8—C9	0.4 (3)
N3—N2—C1—N1	−1.4 (3)	C7—C8—C9—C4	0.7 (3)
N3—C2—C3—N4	174.81 (16)	C9—C4—C5—C6	1.4 (3)
N3—C2—C3—C16	−4.6 (3)	C10—N1—C1—S1	1.7 (4)
N3—C2—C4—C5	−74.1 (2)	C10—N1—C1—N2	−177.6 (2)
N3—C2—C4—C9	105.6 (2)	C10—C11—C12—C13	0.4 (6)
C1—N1—C10—C11	14.2 (4)	C11—C10—C15—C14	−3.9 (5)
C1—N1—C10—C15	−170.8 (3)	C11—C12—C13—C14	−5.6 (6)
C1—N2—N3—C2	177.18 (17)	C12—C13—C14—C15	6.1 (7)
C2—C4—C5—C6	−178.89 (17)	C13—C14—C15—C10	−1.4 (7)
C2—C4—C9—C8	178.75 (16)	C15—C10—C11—C12	4.3 (5)
C3—C2—C4—C5	105.27 (19)		

### Hydrogen-bond geometry (Å, º)

Cg1 is the centroid of the C4–C9 phenyl ring.

**Table:**

*D*—H···*A*	*D*—H	H···*A*	*D*···*A*	*D*—H···*A*
O1—H1···N4^i^	0.82	2.20	2.867 (2)	139
C5—H5···O1^ii^	0.93	2.84	3.451 (3)	124
C5—H5···N4^ii^	0.93	2.85	3.472 (2)	125
C6—H6···O1^ii^	0.93	2.82	3.442 (3)	125
C11—H11···S1	0.93	2.54	3.193 (2)	127
O1—H1···*Cg*1^i^		2.78	3.3309 (17)	126

Symmetry codes: (i) −*x*+3, −*y*+1, −*z*+1; (ii) −*x*+2, −*y*+1, −*z*+1.
