# Supplementary figures and images for: Crystal structure of 2-[2-(hy­droxy­imino)-1-phenyl­propyl­idene]-N-phen­ylhydrazinecarbo­thio­amide
Source: Acta Crystallogr E Crystallogr Commun. 2015 Sep 26;71(Pt 10):o796–7. doi: 10.1107/S2056989015017739 (PMC4647349; doi:10.1107/S2056989015017739)

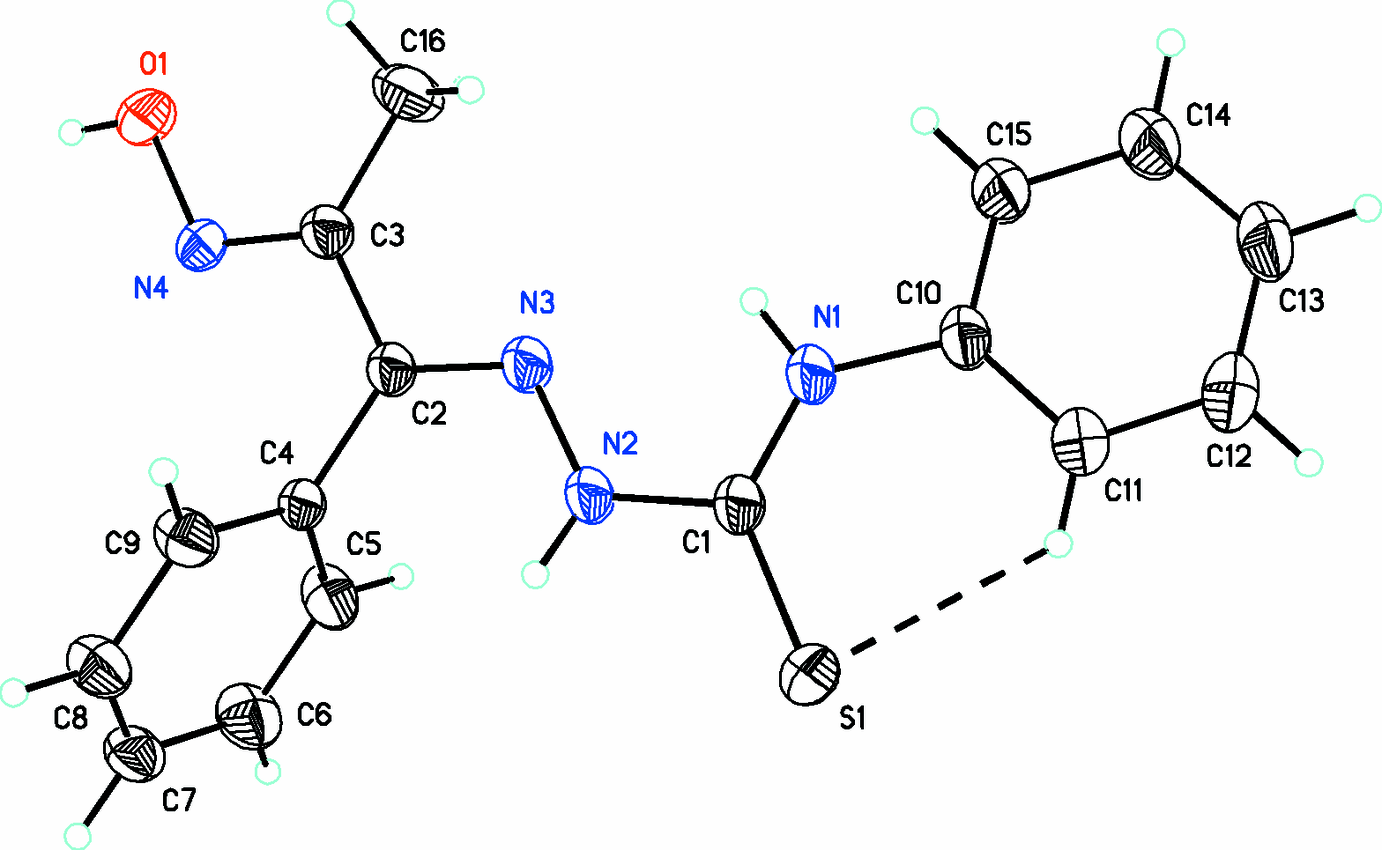

Supplement: Supplementary file 4 [file e-71-0o796-fig1.tif]

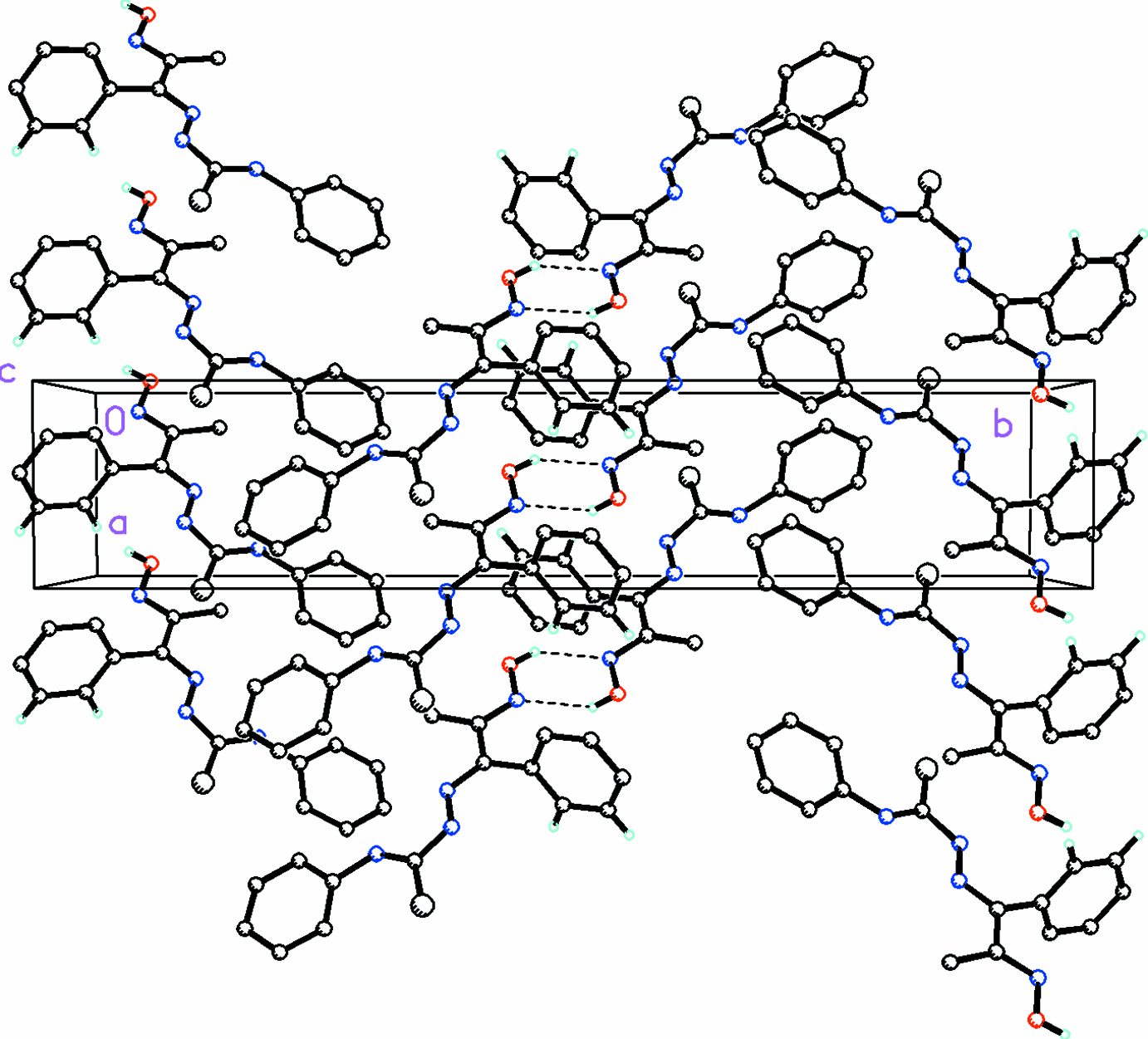

Supplement: Supplementary file 5 [file e-71-0o796-fig2.tif]
